# Characterization and phylogenetic analysis of the complete mitochondrial genome of *Aphis spiraecola* (Hemiptera: Aphididae)

**DOI:** 10.1080/23802359.2019.1673240

**Published:** 2019-10-01

**Authors:** Yimin Du, Xinjun Liu, Ying Wang, Zhanjun Lu

**Affiliations:** a School of Life Sciences, Gannan Normal University, Ganzhou, Jiangxi, China;; b National Navel Orange Engineering and Technology Research Center, Ganzhou, Jiangxi, China

**Keywords:** Aphididae, mitochondrial genome, *Aphis spiraecola*, phylogenetic analysis

## Abstract

*Aphis spiraecola* is an important pest of citrus and transmits a number of plant viruses. Here, we sequenced and annotated the complete mitochondrial genome (mitogenome) of *A. spiraecola*. This mitogenome was 15,465 bp long and encoded 13 protein-coding genes (PCGs), 22 transfer RNA genes (tRNAs), and 2 ribosomal RNA unit genes (rRNAs). Gene order was conserved and identical to that of *Drosophila yakuba* and to most other previously sequenced Aphididae. The whole mitogenome exhibited heavy AT nucleotide bias (81.8%). All 13 PCGs were initiated by the ATN (ATG, ATT and ATA) codon. Except for *cox1* and *nad4* which end with the incomplete codon T−, all PCGs terminated with the stop codon TAA. Phylogenetic analysis showed that *A. spiraecola* got together with the same genus species *Aphis gossypii* and *Aphis craccivora* with high support value, and *Aphis* had a close relationship with *Schizaphis* and *Rhopalosiphum*.

The spirea aphid, *Aphis spiraecola* Patch (Hemiptera: Aphididae), is a pest of citrus and is a vector of citrus tristeza virus (CTV) (Yokomi and Tang 1995; Tsai and Wang 2001). *Aphis spiraecola* originated from eastern Asian origin and has a worldwide distribution, which can also damage apples, ornamentals, and pears (Blackman and Eastop 2000; Cao et al. 2012).

Specimens of *A. spiraecola* were collected from Xinyu City, Jiangxi Province, China (27°52′N, 114°55′E, May 2019) and were stored in Entomological Museum of Gannan Normal University (Accession number GNU-AS039). After morphological identification, total genomic DNA was extracted from tissues using DNeasy DNA Extraction kit (Qiagen, Valencia, CA). Mitogenome sequence was generated using Illumina HiSeq 2500 Sequencing System (Illumina, San Diego, CA). In total, 5.7 G raw reads were obtained, quality-trimmed and assembled using MITObim v 1.7 (Hahn et al. 2013). By comparison with the homologous sequences of other Aphididae species from GenBank, the mitogenome of *A. spiraecola* was annotated using software GENEIOUS R8 (Biomatters Ltd., Auckland, New Zealand).

The complete mitogenome of *A. spiraecola* is 15,465 bp (Genbank accession, MN316642). It contains 13 protein-coding genes (PCGs), 22 tRNA genes, 2 rRNA genes, and a non-coding AT-rich region. Gene order was conserved and identical to that of *Drosophila yakuba* and to most other previously sequenced Aphididae (Thao et al. 2004; Ren et al. 2016; Li et al. 2017; Chen et al. 2019). The nucleotide composition of the mitogenome was biased toward A and T, with 81.8% of A + T content (A 45.1%, T 38.9%, C 10.2%, G 5.8%). Of the 13 PCGs, 4 PCGs (*nad4*, *nad4l*, *nad5*, and *nad1*) were encoded by the minority strand (N-strand) while the other nine were located on the majority strand (J-strand). All PCGs started with the standard ATN codons (eight ATT, three ATA, and two ATG). Most of the PCGs terminated with the TAA stop codon, while an incomplete stop codon T − was found in two genes (*cox1* and *nad4*). The 22 tRNA genes vary from 62 bp (*trnW*, *trnT*, and *trnV*) to 73 bp (*trnK*). Two rRNA genes (*rrnL* and *rrnS*) locate at *trnL1*/*trnV* and *trnV*/control region, respectively. The lengths of *rrnL* and *rrnS* in *A. spiraecola* are 1263 and 775 bp, with the AT contents of 85.4 and 84.5%, respectively.

Phylogenetic tree was constructed using the maximum-likelihood method through raxmlGUI 1.5 (Silvestro and Michalak 2012) based on 13 mitochondrial protein-coding genes sequences (Figure 1). Results showed that the new sequenced species *A. spiraecola* got together with the same genus species *Aphis gossypii* and *Aphis craccivora* with high support value (BS = 97), and *Aphis* had a close relationship with *Schizaphis* and *Rhopalosiphum*. In conclusion, the mitogenome of *A. spiraecola* is obtained in this study and can provide essential and important DNA molecular data for further phylogenetic and evolutionary analysis of Aphididae.

**Figure 1.**
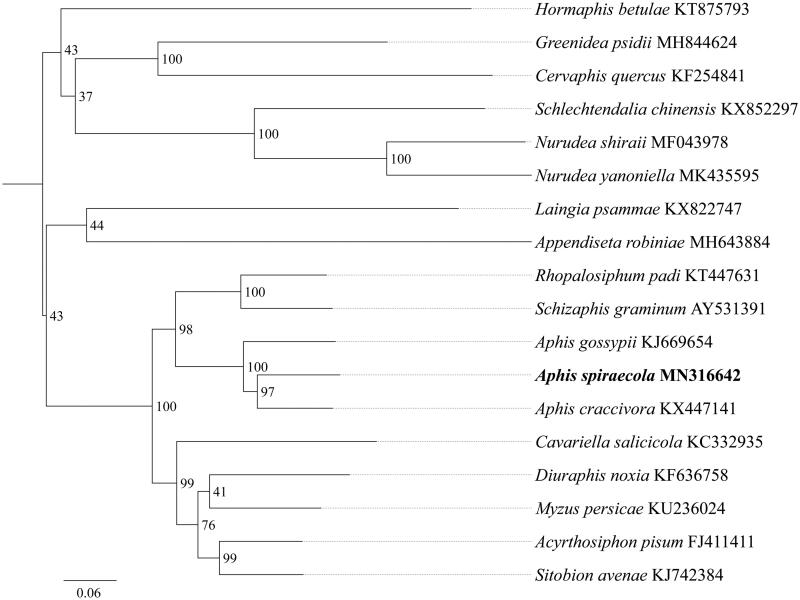
Phylogenetic relationships based on the 13 mitochondrial protein-coding genes sequences inferred from RaxML. Numbers on branches are Bootstrap support values (BS).

## References

[CIT0001] Blackman RL, Eastop VF 2000 Aphids on the world’s crops: an identification and information guide. 2nd ed. New York (NY): John Wiley & Sons Ltd.

[CIT0002] Cao J, Li J, Niu J, Liu X, Zhang Q 2012 Population structure of *Aphis spiraecola* (Hemiptera: Aphididae) on pear trees in China identified using microsatellites. J Econ Entomol. 105:583–591. 22606830 10.1603/ec11368

[CIT0003] Chen J, Wang Y, Qin M, Jiang LY, Qiao GX 2019 The mitochondrial genome of *Greenidea psidii* van der Goot (Hemiptera: Aphididae: Greenideinae) and comparisons with other Aphididae aphids. Int J Biol Macromol. 122:824–832. 30389524 10.1016/j.ijbiomac.2018.10.209

[CIT0004] Hahn C, Bachmann L, Chevreux B 2013 Reconstructing mitochondrial genomes directly from genomic next-generation sequencing reads-a baiting and iterative mapping approach. Nucleic Acids Res. 41:e129. 23661685 10.1093/nar/gkt371 PMC3711436

[CIT0005] Li YQ, Chen J, Qiao GX 2017 Complete mitochondrial genome of the aphid *Hormaphis betulae* (Mordvilko) (Hemiptera: Aphididae: Hormaphidinae). Mitochondrial DNA Part A. 28:265–266. 10.3109/19401736.2015.1118071 26713493

[CIT0006] Ren ZM, Bai X, Harris A, Wen J 2016 Complete mitochondrial genome of the *Rhus* gall aphid *Schlechtendalia chinensis* (Hemiptera: Aphididae: Eriosomatinae). Mitochondrial DNA Part B. 1:849–850. 10.1080/23802359.2016.1241678 PMC7799637 33473653

[CIT0007] Silvestro D, Michalak I 2012 RaxmlGUI: a graphical front-end for RAxML. Org Divers Evol. 12:335–337.

[CIT0008] Thao ML, Baumann L, Baumann P 2004 Organization of the mitochondrial genomes of whiteflies, aphids, and psyllids (Hemiptera, Sternorrhyncha). BMC Evol Biol. 4:25. 15291971 10.1186/1471-2148-4-25 PMC512530

[CIT0009] Tsai JH, Wang JJ 2001 Effects of host plants on biology and life table parameters of *Aphis spiraecola* (Homoptera: Aphididae). Environ Entomol. 30:44–50.

[CIT0010] Yokomi R, Tang Y 1995 Host preference and suitability of two aphelinid parasitoids (Hymenoptera: Aphelinidae) for aphids (Homoptera: Aphididae) on citrus. J Econ Entomol. 88:840–845.

